# How to estimate body condition in large lizards? Argentine black and white tegu (*Salvator merianae*, Duméril and Bibron, 1839) as a case study

**DOI:** 10.1371/journal.pone.0282093

**Published:** 2023-02-24

**Authors:** Kelly R. McCaffrey, Sergio A. Balaguera-Reina, Bryan G. Falk, Emily V. Gati, Jenna M. Cole, Frank J. Mazzotti

**Affiliations:** 1 Department of Wildlife Ecology and Conservation, Fort Lauderdale Research and Education Center, University of Florida, Davie, Florida, United States of America; 2 South Florida Natural Resources Center, National Park Service, Homestead, Florida, United States of America; University of Veterinary Medicine Vienna: Veterinarmedizinische Universitat Wien, AUSTRIA

## Abstract

Body condition is a measure of the health and fitness of an organism represented by available energy stores, typically fat. Direct measurements of fat are difficult to obtain non-invasively, thus body condition is usually estimated by calculating body condition indices (BCIs) using mass and length. The utility of BCIs is contingent on the relationship of BCIs and fat, thereby validation studies should be performed to select the best performing BCI before application in ecological investigations. We evaluated 11 BCIs in 883 Argentine black and white tegus (*Salvator merianae*) removed from their non-native range in South Florida, United States. Because the length-mass relationship in tegus is allometric, a segmented linear regression model was fit to the relationship between mass and length to define size classes. We evaluated percent, residual, and scaled fat and determined percent fat was the best measure of fat, because it was the least-associated with snout-vent length (SVL). We evaluated performance of BCIs with the full dataset and within size classes and identified Fulton’s K as the best performing BCI for our sampled population, explaining up to 19% of the variation in fat content. Overall, we found that BCIs: 1) maintained relatively weak relationships with measures of fat and 2) splitting data into size classes reduced the strength of the relationship (i.e., bias) between percent fat and SVL but did not improve the performance of BCIs. We postulate that the weak performance of BCIs in our dataset was likely due to the weak association of fat with SVL, the body plan and life-history traits of tegus, and potentially inadequate accounting of available energy resources. We caution against assuming that BCIs are strong indicators of body condition across species and suggest that validation studies be implemented, or that alternative or complimentary measures of health or fitness should be considered.

## Introduction

Body condition is a relevant measure commonly used to gauge individual and population-level health in ecology, wildlife management, and conservation biology [1–3]. Measures of body condition are used to assess the effect of biotic and abiotic factors on the health of individuals or populations [4] and has been related to aspects of individual fitness such as survival, reproductive success, and predator-prey dynamics [2, 5–10]. Nonetheless, the body condition of an individual, as measured by physical energy reserves which impact fitness (e.g., fat), can be difficult to quantify and often requires the sacrifice of the study organism. Thus, the relationship between body mass (M) and length (L) is commonly used to calculate body condition indices (BCIs) in a particular species to represent their relative condition [11–13]. Generally, the relationship between M and L is modeled by the general allometric equation (*M* = *aL^b^*) with parameters *a* (intercept) and *b* (slope) commonly estimated by linear regression based on the log-transformed equation ($\log_{e}\;M=\log_{e}\;a+b\;\log_{e}\;L$).

Commonly used BCIs in ecological and management studies can be categorized as ratio or residual BCIs [12] (Table 1). Ratio BCIs have been the longest used to measure relative “fatness” by taking the ratio of M to L or L raised to a specific power ($a=\frac{M}{L^{b}}$) [4, 11, 12, 14–17]. These BCIs have the benefit of being relatively simple, easy to interpret and communicate, and are comparable across populations since they do not rely on population-specific parameters (e.g., mean L of population). However, ratio BCIs are often correlated with body size, limiting the comparisons of ratio BCIs to animals of similar sizes [4, 11]. Some ratio BCIs such as Fulton’s K ($\frac{M}{L^{3}}$) assume that the slope of the log-transformed relationship between mass and length is exactly 3.0 (*b* is exactly 3.0) and comparisons between populations may give erroneous results if *b* is significantly different, even if the mean size of the individuals in each population is the same [4].

**Table 1:** Summary of the body condition indices (BCIs) used in this study. Naming and abbreviations follow Falk et al. [3]. Additional abbreviations indicating regression types are defined as follows: standard major axis (SMA), major axis (MA), and ordinary least squares (OLS).

Name	Abbreviation	Description	Category	Citations
Ratio index	M/L	Body mass divided by body length	Ratio	[8]
Quételet index	M/L^2^	Body mass divided by body length squared	Ratio	[15]
Fulton’s index	M/L^3^	Body mass divided by body length cubed	Ratio	[16, 17]
Relative index	M/prM	Body mass divided by predicted body mass from SMA regression	Ratio	[14]
Log ratio index	logM/logL	Ln-transformed body mass divided by ln-transformed body length	Ratio	[8]
Log relative index	logM/log(prM)	Ln-transformed body mass divided by predicted body mass from SMA regression	Ratio	[1]
OLS residual index	OLSres	Residuals from OLS regression of ln-transformed body mass on ln-transformed body length	Regression (Type I)	[11]
MA residual index	MAres	Residuals from MA linear regression of ln-transformed body mass on ln-transformed body length	Regression (Type II)	[12, 18]
SMA residual index	SMAres	Residuals from SMA linear regression of ln-transformed body mass on ln-transformed body length	Regression (Type II)	[12, 18]
Cubed regression index	res(M~L^3^)	Residuals from SMA linear regression of body mass on body length cubed	Regression (Type II)	[13]
Scaled mass index	SMI	Scaled Mass Index	Allometric	[19]

Residual indices are a popular alternative to ratio indices because they are less prone to being associated with body size [11, 12]. To calculate residual BCIs, residuals are taken from the linear regression of M on L. Positive values indicate an individual which has a higher mass than predicted by the linear regression based on length, while negative values indicate an individual which has a lower mass than expected [18]. Appropriate application of residual BCIs requires that several key assumptions be met, including: 1) data must be linear, 2) the variance is homoscedastic, and 3) the residuals are normally distributed [12]. Additionally, residual BCIs are not comparable across populations in time and space since the regression slope *b* may differ [11].

The scaled mass index (SMI) has been proposed more recently as an alternative BCI, which is better suited to deal with allometric changes in shape as animals grow [19, 20]. The SMI standardizes body mass for a predefined value of body size according to the Thorpe-Lleonart (TL) scaling model: $SMI=M_{i}{\left(\frac{L_{0}}{L_{i}}\right)}^{b_{SMA}}$, where M_i_ is the body mass of the *i*^th^ individual, L_i_ is the body size of the *i*^th^ individual, L_0_ is a predefined value of body size and b_SMA_ is the scaling exponent calculated by a standard major axis (SMA) regression of ln-transformed body mass on ln-transformed body size [21]. Thus, SMI is the predicted mass for individual *i* after correcting for the scaling relationship between body size (length) and mass, removing confounding effects of allometry. Comparability between populations is also improved from other BCIs as b_SMA_ and L_0_ may be standardized and SMI does have the same set of assumptions as residual BCIs, which are commonly violated [19, 20].

The standard allometric mass-length equation is generally assumed to fit over the total of the population being sampled but may not fit well when applied to a large range of body sizes, leading to consistent under- or over-estimates of weight at certain lengths [22] and biased estimates of residual and ratio BCIs. Alternatively, it may be appropriate to fit alternative weight-length models for different size groups or age classes [22]. Finally, body condition indices assume that energy reserve mass is independent of length [12, 18], and thus the relationship between measures of fat and length being used in body condition studies for a species should be evaluated.

Many reptiles have paired coelomic fat bodies which serve as a primary location for fat storage [23]. In snakes, these abdominal fat bodies have been shown to be good representatives of total lipid content in the body [24]. Compared to snakes, lizards may have additional appendages, such as four limbs and an elongate tail, which could potentially influence the performance of BCIs. Many lizards, including the Argentine black and white tegu (*Salvator merianae*, Duméril and Bibron, 1839), have additional stores of adipose tissue in the tail and within the liver [23, 25] and tegus undergo seasonal fat cycles related to both reproduction and brumation [25, 26], making interpretation of reliable BCIs more challenging. We used coelomic wet-fat mass of tegus to approximate true body condition, because measuring total fat mass by drying and grinding specimens to perform chemical extraction of lipids [24] was not feasible given the size and number of specimens included in this study. The mass of abdominal fat bodies represents a large proportion of total body lipids in tegus [25] and has been previously shown to fluctuate in a similar way to total lipid content in other squamates [24]. The coelomic wet-fat mass of tegus has also been used in other investigations to evaluate seasonal cycles related to reproduction and brumation [25, 27, 28]. Because of its previous links to physiological cycles in tegus, and because it was not feasible to perform total lipid extractions, we believe that the use of abdominal fat bodies is a suitable alternative measure of true body condition.

Herein, we evaluated the performance of 11 BCIs (ratio, residual, and SMI) in a non-native population of Argentine black and white tegus in South Florida, United States. We used measures of body mass and snout-vent length collected during necropsy to calculate BCIs with the objective of understanding the reliability of those values to gauge individual and population-level energy stores. We removed and weighed the discrete fat bodies in the coelomic cavity during necropsy to obtain wet-fat mass and calculate three alternative measures of fat stores (percent fat, scaled fat, and residual fat) as representatives of energy stores. We asked which measure of fat stores was best for validating BCIs, which BCIs best approximated fat stores, whether BCIs were associated with body size, and how BCIs related to one another. Finally, we characterized the allometric relationship between mass and length for tegus in our study to define size class groups. We analyzed our dataset as a whole and additionally investigated the impact of applying measures of body condition within the defined size classes of the dataset to evaluate the effect on BCI performance.

## Materials and methods

### Species description

The Argentine black and white tegu is a large teiid lizard native to eastern and central South America that has become established in several areas of central and southern Florida, United States [27, 29–31]. Tegus can be classified as omnivores as well as generalist meso-predators which consume fruit, plant material, and animal prey (arthropods, gastropods, reptiles, birds, and small mammals) [32–34], with evidence of diet changing based on seasonal abundance [34]. Tegus are also known to scavenge upon carrion and regularly consume eggs of ground-nesting vertebrates such as alligators, turtles, and birds [33–35]. This species is considered invasive in Florida with potential negative impacts on native fauna through egg predation and competition for burrowing sites [35]. To combat this threat, management programs have been established in Florida to reduce the population of invasive tegus and halt their expansion into ecologically sensitive areas, such as nesting sites of threatened species such as American crocodile (*Crocodylus acutus*) and gopher tortoises (*Gopherus polyphemus*) [30, 31].

### Collection, euthanasia, and necropsy

We received tegus collected through trapping and removal efforts performed by the Florida Fish and Wildlife Conservation Commission (FWC) and the University of Florida (UF) from 2012 through 2018. Once obtained, tegus were humanely euthanized using captive bolt or firearm immediately followed by pithing, and frozen until necropsy. Tegus were thawed prior to necropsy and examined for general health and condition by visually inspecting all internal organs and the body exterior for any abnormalities or deformities that may affect body mass, body length, or fat mass. We obtained measurements of snout-vent length (SVL) to the nearest 0.1 cm using a flexible measuring tape, and total body mass using a digital scale to the nearest g. Coelomic wet-fat mass was obtained by removing and weighing the discrete abdominal fat bodies to the nearest 0.0001 g. The average timespan a tegu was held between euthanasia and necropsy was approximately 263 ± 258 days (range 0–1,847 days). The research protocol was approved by the University of Florida Animal Research Committee and University of Florida Institutional Animal Care and Use Committee and protocol numbers are listed in the Acknowledgements.

### Statistical methods

To reduce potential bias in our dataset, we excluded data from tegus with incomplete or unreliable necropsies due to decay, unknown sex, physical abnormalities (missing or abnormal limbs or tails (e.g., regenerated tail), and scoliosis), or if the time spent in captivity prior to euthanasia was more than 4 days. We also removed animals whose wet-fat mass was equal to 0, which could not be transformed with natural log. All data analyses were performed in R [36].

To determine whether sex influenced the relationship between wet-fat mass, body mass, and SVL, we performed ln-transformed standard major axis (SMA) regressions of wet-fat mass and body mass on SVL and used the likelihood ratio test for common slopes via the “smatr” package [37] to assess for differences in the slope of the regression line between sexes [38]. Finally, we performed a Kendall’s τ correlation test to determine whether the number of days an animal was held between euthanasia and necropsy was associated with SVL, total mass, wet-fat mass, or the 11 BCIs.

### Size class definition

We examined the relationship between raw total mass and SVL measurements in the clean dataset to detect inflection points (changes in slope) and fit a segmented linear regression onto the allometric curve via the “segmented” package [39, 40]. We initialized the model by fitting a generalized linear model with a Gamma distribution and negative inverse link to the relationship between total mass and SVL for the full dataset. The most-parsimonious number of breakpoints in this initial relationship and their values were estimated using the Bayesian information criterion (BIC) with Bonferroni p-value correction [40], testing for 0 to 10 breakpoints. The fit of segmented linear regressions with different breakpoints, up to 10, were compared to each other and to the fit of the basic allometric model of the data with no breakpoints, to determine if a segmented model fit the data better than the basic allometric model. We did this to define groups within the dataset that relate isometrically, in an attempt to avoid violating critical assumptions (linear relationship between ln-transformed mass and ln-transformed SVL) of the BCIs being tested [12, 22, 41] and improve the performance of BCIs by using smaller size groups [22].

After detecting the optimum number of breakpoints, a three-segmented linear model was fit to the data and size classes were assigned to tegus in the dataset. Finally, we created a graph to visualize the fit of the resulting segmented linear regression with the fit of a basic allometric model of the data [4, 22]. We took the mean and standard deviation of tegu weights in groups with ranges of 2.0 cm, for tegus with SVLs between 10.0 cm to 46.4 cm. We plotted the mean weight of each size-class group, with error bars representing the standard error, and compared the overlap of the basic allometric model and the segmented linear regression, expecting that the best-fitting model would better-intersect the mean weights upon visual inspection (S1 Fig).

### Body condition assessment

To understand the effect of analyzing data by the size groups defined by SVL using the segmented linear regression, compared to analyzing it as a whole, the following analyses were done twice, first including all the data and after by size class. We calculated percent fat, scaled fat, and residual fat for each tegu as alternative measures of fat stores to validate the various BCIs. Percent fat was calculated by dividing the coelomic wet-fat mass by the total mass. We calculated scaled fat via SMI [19] with wet-fat mass and SVL and using mean SVL as L_0_. We calculated residual fat by performing an ln-transformed SMA regression of wet-fat mass on SVL and taking the residuals of the model. When analyzing the data by size group, we calculated SMI, scaled fat, and residual fat for each group individually using that group’s L_0_ and SMA regression model residuals. We estimated the slopes and adjusted r^2^ values from OLS regressions of each of the measures of fat stores (i.e., percent fat, scaled fat, and residual fat) on ln-transformed SVL, because ideally, measures of stored fat should not be associated with body length assuring independence between variables. Fat-store measures that are not associated with body length will have non-significant slopes and small adjusted r^2^ values.

BCIs were calculated using body mass and length (SVL) measurements. We tested the assumptions of residual BCI measures, including that the data are linear, the variance is constant (i.e., homoscedastic), and the residuals are normally distributed [12]. We tested for linearity using Ramsey’s RESET test [42], for non-constant variance using the Breusch-Pagan test [43] and normality of residuals using the Shapiro-Wilk test [44]. For residual BCIs which deviated from normality, we tested for skewness using the D’Agostino test [45] and for kurtosis using the Anscombe-Glynn test [46].

We gauged the performance of BCIs by testing the association between each BCI and our three measures of fat as well as SVL using Kendall’s correlation test [47] to find Kendall’s τ. We also performed OLS linear regressions of BCIs on the three measures of fat stores (to evaluate their performance as a BCI) and SVL (to test for a size bias), calculating adjusted r^2^ values to estimate the proportion of variation in each BCI that could be explained by measures of fat stores and SVL. We created a correlation matrix among BCIs by calculating pairwise Kendall rank correlation coefficients (τ) focused on understanding the strength of association between BCIs to determine which were strongly correlated and could be substituted for each other. Dispersion values are reported as standard error (SE) and 95% confidence intervals (CI).

## Results

**Fig 1:**
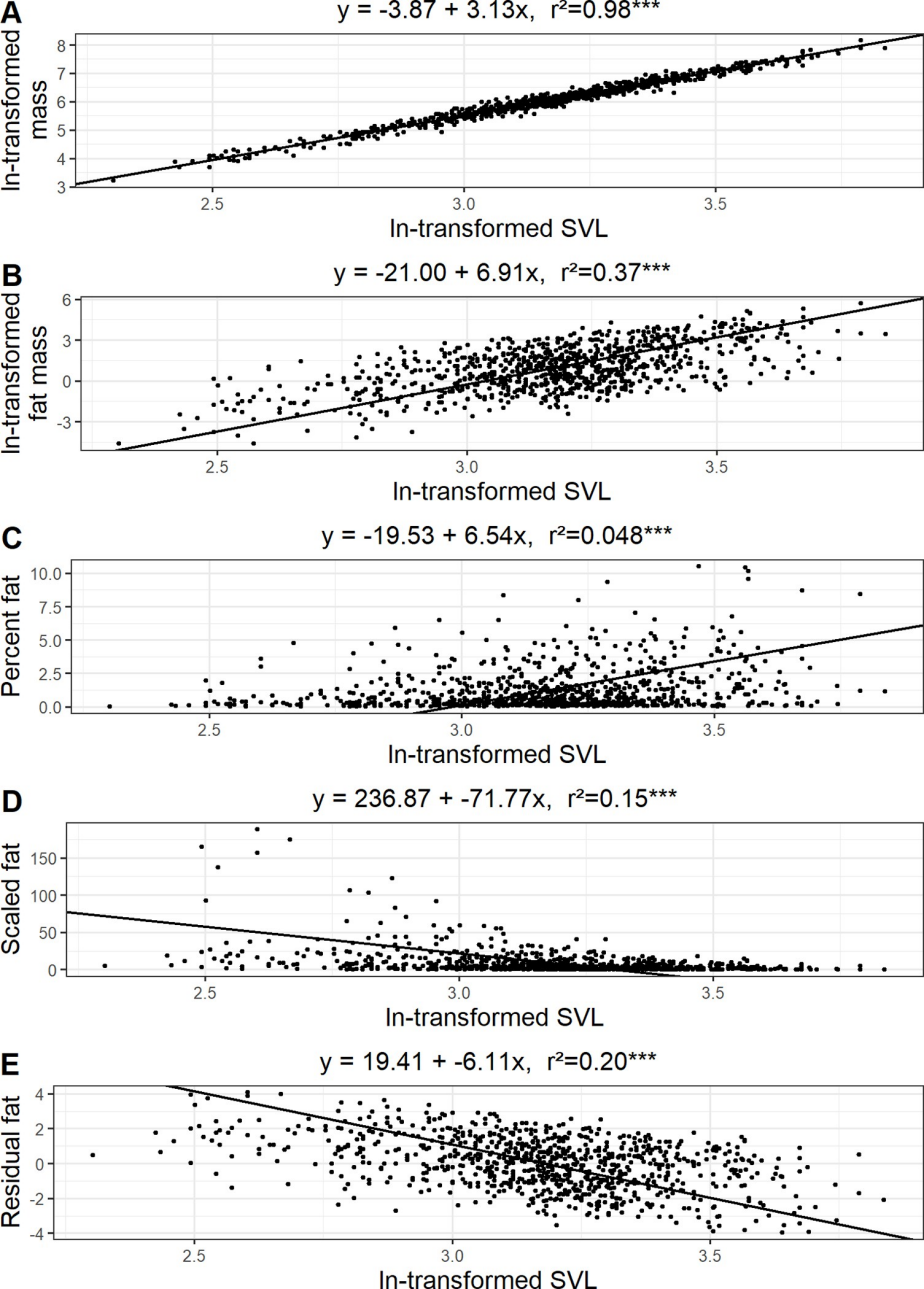
Relationships between ln-transformed snout-vent length (SVL). and A) ln-transformed body mass, B) ln-transformed wet-fat mass, C) percent fat, D) scaled fat, and E) residual fat. SMA regression equations intercepts, slopes, and adjusted r^2^ values are reported to two significant digits. Significance values are reported as * P ≤ 0.05, ** P ≤ 0.01, and *** P ≤ 0.001.

We collected and processed a total of 2,658 tegus between 20^th^ February 2012 and 26^th^ October 2018 mainly from the Southern Glades in Homestead, Florida. However, only 883 tegus (496 males and 387 females) met all the requirements to be used for analysis (Table 2). Shapiro-Wilk tests showed that the morphometric variables within our dataset (SVL, body mass, and wet-fat mass) were not normally distributed (p-values all < 0.001). Mann-Whitney U tests showed no significant difference in SVL (p-value = 0.69) or total body mass (p-value = 0.78) between sexes, but females had a significantly greater wet-fat mass (p-value = 0.008; Table 2). In general, tegus maintained relatively small coelomic fat stores, averaging only 1.3 ± 0.05% of total mass. However, percent fat ranged an order of magnitude, with a maximum of about 10.5% of the total body mass (Table 2). The ln-transformed SMA regressions showed that tegus exhibited positive allometry in the relationship between body mass and SVL with a slope equal to 3.13 (CI: 3.09–3.16, Fig 1A). Tegus also exhibited positive allometry in the relationship between wet-fat mass and SVL with a slope equal to 6.91 (CI: 6.56–7.28; Fig 1B), though the adjusted r^2^ value was low (r^2^ = 0.37). Ramsey’s RESET test showed that the relationship between ln-transformed body mass and ln-transformed SVL and ln-transformed fat mass and ln-transformed SVL were both linear (RESET = 3.54 and 0.09, p-value = 0.06 and 0.76, respectively).

**Table 2:** Summary of morphometric and fat measurements from Argentine black and white tegus included in this study. Values are expressed as mean ± standard error (range). Size groups (1, 2, and 3) were defined based on the fitted segmented regression model (Fig 2), which defined size groups as hatchlings to early juveniles (group 1, < 20.2 cm SVL), late juveniles to reproductive-sized adults (group 2, 20.2 cm to 30.0 cm SVL) and large adults (group 3, > 30.0 cm SVL).

Sex or Size Group	N	SVL (cm)	Total mass (g)	Wet-fat mass (g)	Percent fat (%)	Scaled fat	Residual fat
Female	387	24.4 ± 0.3 (11.4–38.2)	531.9 ± 17.6 (39.9–1,960.0)	10.3 ± 1.0 (0.02–171.4)	1.4 ± 0.1 (0.02–10.5)	8.7 ± 0.7 (0.09–156.7)	0.2 ± 0.1 (-3.5–3.9)
Male	496	24.9 ± 0.3 (10.0–46.4)	582.7 ± 21.0 (25.0–3,500.0)	8.3 ± 0.9 (0.01–296.4)	1.1 ± 0.1 (0.01–9.6)	8.9 ± 0.9 (0.1–188.3)	-0.2 ± 0.1 (-4.0–4.1)
1	180	17.0 ± 0.2 (10.0–20.1)	158.2 ± 4.9 (25.0–320.0)	1.8 ± 0.2 (0.01–16.3)	1.0 ± 0.1 (0.01–6.5)	2.3 ± 0.4 (0.01–37.9)	0 ± 0.1 (-4.0–4.0)
2	558	24.8 ± 0.1 (20.2–29.9)	497.7 ± 7.1 (210.0–1,050.0)	6.3 ± 0.4 (0.1–72.0)	1.2 ± 0.1 (0.02–9.4)	9.4 ± 0.7 (0.1–157.6)	0 ± 0.1 (-3.8–4.0)
3	145	34.0 ± 0.3 (30.0–46.4)	1,301.1 ± 36.8 (550.0–3,500.0)	29.7 ± 3.4 (0.5–296.4)	2.1 ± 0.2 (0.04–10.5)	42.2 ± 4.8 (0.1–293.9)	0 ± 0.2 (-4.6–3.1)

The likelihood ratio test for common slopes analysis showed that the allometric relationship between ln-transformed body mass and ln-transformed SVL did not significantly differ between males and females (p-value = 0.57). However, the allometric relationship between ln-transformed wet-fat mass and ln-transformed SVL differed significantly between males and females (p-value = 0.02) with a greater predicted slope value for females (7.38) than males (6.55) but overlapping confidence intervals (females 6.85–7.95, males 6.55–7.04). Due to the overlapping confidence intervals of the slope estimates, the difference between sexes appeared to be small and the sexes were not separated for further analyses.

Each of our measures of fat stores was associated with SVL to some degree (Fig 1C–1E). Of the transformed measures of fat, residual fat exhibited the strongest relationship with SVL suggesting that SVL explained approximately 20% of the variation in residual fat (Fig 1E). Both residual fat and scaled fat had significant negative relationships with SVL. The relationship between percent fat and SVL was significant and positive but weak, with SVL explaining approximately 5% of the variation in percent fat (Fig 1C). Percent fat is the best measure of true body condition in this case, because it is the most weakly correlated with SVL.

### Size-class groups definition

**Fig 2:**
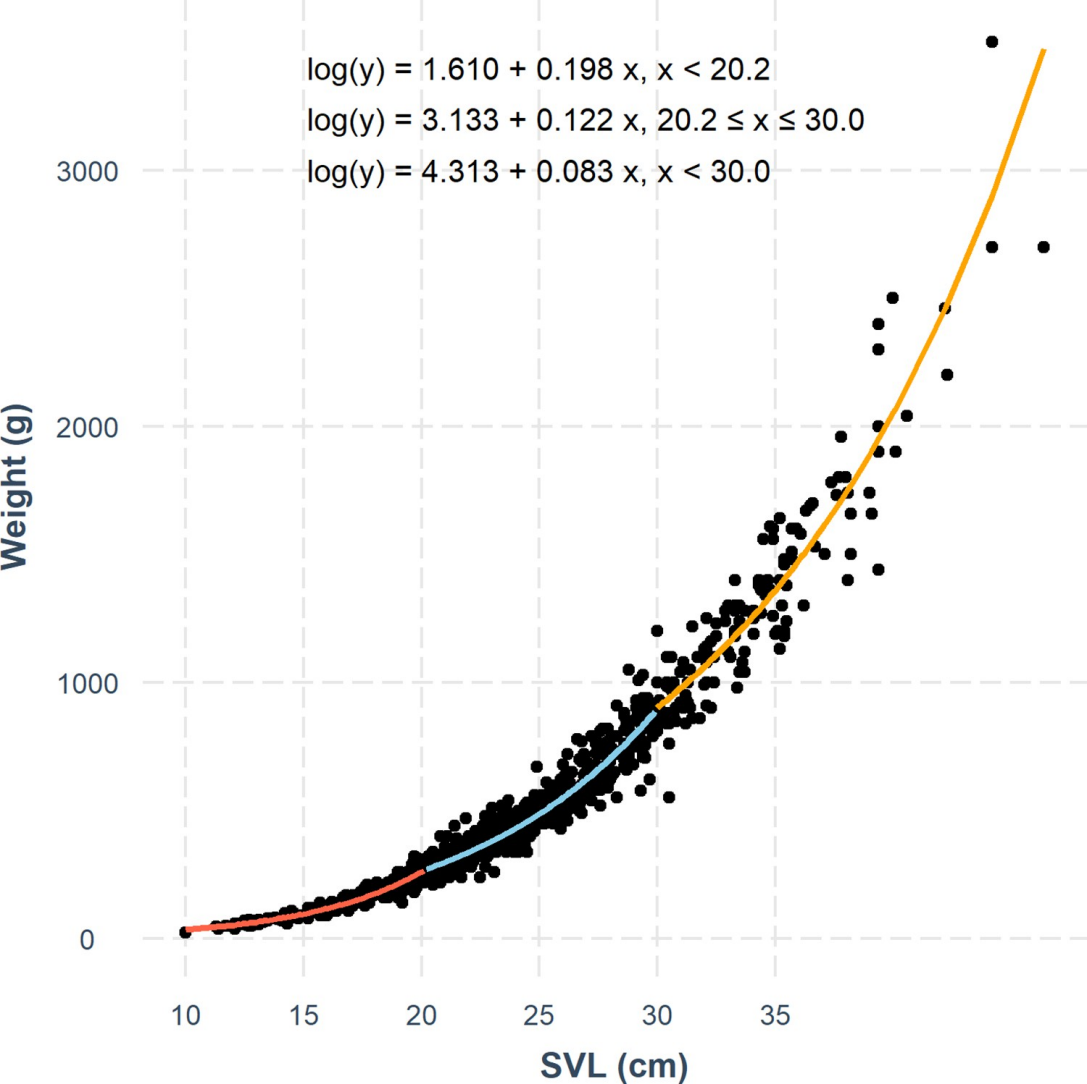
The three-segmented generalized linear model fit to the relationship between snout-vent length (SVL) and total mass, splitting the data into three size groups: Hatchlings to early juveniles (group 1, < 20.2 cm), late juveniles to reproductive-sized adults (group 2, 20.2 cm– 30.0 cm) and large adults (group 3, > 30.0 cm).

Based on our data, the optimal number of breakpoints in a segmented linear regression model of total body mass to SVL were 20.2 ± 0.2 cm and 30.0 ± 0.5 cm, splitting the data into three size groups: hatchings to early juveniles < 20.2 cm, late juveniles to reproductive-sized adults between 20.2 and 30.0 cm and large adults > 30.0 cm (Fig 2). In most cases, the segmented linear regression model intersected well with the actual data and seemed to fit as well or better than the standard allometric model for mid-sized tegus. Model segments representing the smallest and largest tegus had few estimates that departed from the actual data or did not fit as well as the standard allometric model (S1 Fig).

### Body condition assessment

#### All data

We found significant but weak correlations between the number of days an animal was held between euthanasia and necropsy, and total body mass (τ = -0.11, p < 0.001), SVL (τ = -0.14, p < 0.001), and wet-fat mass (τ = -0.09, p < 0.001) as well as all 11 BCIs tested (τ = -0.10–0.19, all p-values < 0.05; S1 Table in S1 File). Because of the weak strength of these associations (|τ| ≤ 0.19) we believe it is unlikely that they would significantly influence our results.

The testable assumptions of the regression BCIs were generally not met (p-value < 0.05; Table 3). Homoscedasticity was rejected for the ln-transformed linear regression of body mass on length and the regression of body mass on length cubed. The normality of residuals was rejected for all regression BCIs. Deviations from normality appeared to be driven by skewness and kurtosis for the OLS residual index and cubed regression index, and kurtosis alone in the MA and SMA residual indices (Table 3). The slope assumption of Fulton’s K index (the slope of the relationship between ln-transformed mass and SVL is equal to 3.0; Cone 1989), was also violated (slope = 3.13, CI 3.09–3.16).

**Table 3:** Tests of the assumptions of regression-based body condition indices. We tested three assumptions, whether 1) data are linear, 2) variance is constant (i.e., homoscedastic), and 3) frequency distribution of residuals are normal. Only p-values are reported.

BCI	Regression	Ramsey’s RESET Test (linearity)	Breusch-Pagan Test (homoscedasticity)	Shapiro-Wilk Test (normality)	D’Agostino Test (skewness)	Anscombe-Glynn Test (kurtosis)
OLSres	OLS(logM~logL)	0.060	0.021	0.002	0.074	0.001
Mares	MA(logM~logL)	-	-	0.009	0.221	0.002
SMAres	SMA(logM~logL)	-	-	0.005	0.141	0.001
res(M~L3)	SMA(M~L3)	0.074	<0.001	<0.001	<0.001	<0.001

Across all BCIs tested, adjusted-r^2^ values from linear regressions of BCIs on fat stores ranged between 0.03–0.26, suggesting that at best ~26% of the variation in BCIs could be explained by fat stores (S2 Table in S1 File). Kendall’s correlation coefficient (τ) calculated for BCIs with fat store measures were also low, with absolute values of τ ranging between 0.12–0.36 (S3 Table in S1 File). All BCI measures, except for the OLS residual index, were correlated with SVL. Significant adjusted r^2^ values from the linear regressions of BCIs on SVL ranged between 0.01–0.85 (S2 Table in S1 File) and significant absolute values of τ ranged between 0.06–0.88 (S3 Table in S1 File).

In general, the residual indices, SMI, and Fulton’s K had weaker associations with SVL than other ratio indices. Of the ratio indices, only Fulton’s K maintained positive associations with all measures of fat (S2 and S3 Tables in S1 File). Using percent fat as the measure of true body condition, the best performing BCIs in our sampled population were Fulton’s K (r^2^ = 0.18, τ = 0.33) and the OLS residual index (r^2^ = 0.14, τ = 0.31), due to their high adjusted r^2^ and τ values and their weak or insignificant associations with SVL (Fulton’s K r^2^ = 0.03, τ = 0.10, OLS insignificant r^2^ and τ; S2 and S3 Tables in S1 File). However, it is noted that assumptions of homoscedasticity and normality for the OLS residual index were violated (Table 3). The SMI was the next most-suitable alternative as it does not rely on the assumptions of residual indices being met and is somewhat weakly associated with SVL compared to the other available indices (S2 and S3 Tables in S1 File).

**Fig 3:**
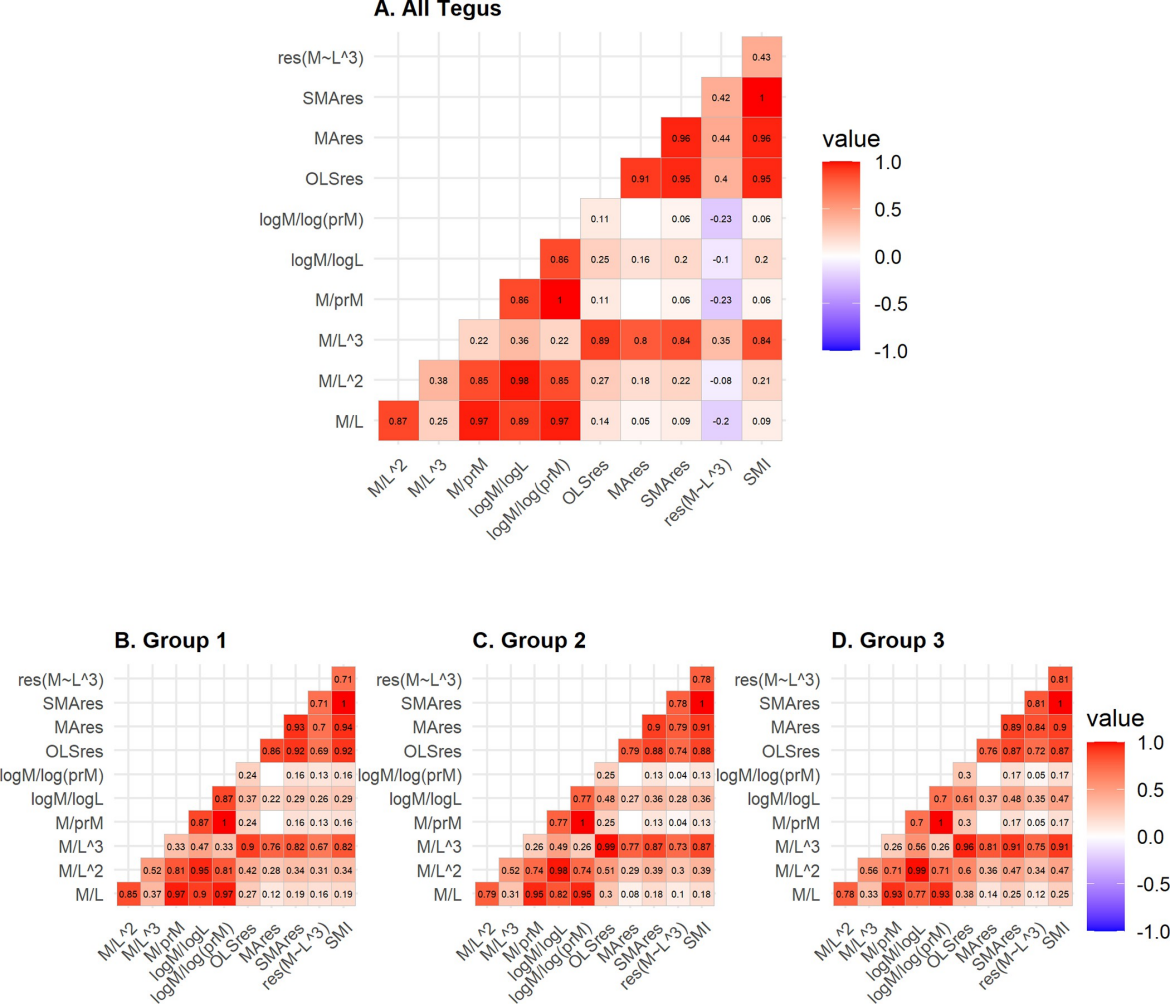
Correlation (Kendall’s τ) among 11 BCIs used in this study, for all tegu (A) and by size class (B-D). Correlations which were insignificant have no displayed τ value.

Ratio BCIs were more strongly correlated with other ratio indices and residual BCIs were more strongly correlated with other residual indices, with a few exceptions (Fig 3). Fulton’s K index maintained a stronger correlation with residual indices (τ = 0.35–0.89) than other ratio indices (τ = 0.22–0.38). Likewise, the SMI was more strongly correlated with residual BCIs and Fulton’s K (τ = 0.43–1) than the ratio indices (τ = 0.06–0.21). The cubed regression index maintained weak, negative correlations with all ratio indices except for Fulton’s K (τ = -0.08 –-0.23) and was relatively weakly, positively correlated with other residual indices as well as the SMI and Fulton’s K (τ = 0.35–0.44).

#### By size groups

When tegus were split into smaller size groups, the relationship between ln-transformed body mass on SVL was nonlinear for the second size group (p-value = 0.026, Table 4), though examination of the plotted variables seems to indicate this violation of linearity is not major (S2 Fig). All size groups exhibited positive allometry in body mass, with the slope of the ln-transformed SMA regressions of body mass on SVL ranging between 3.18–3.26 (S3 Fig). All size groups also exhibited positive allometry in wet-fat mass, with the slope of the ln-transformed SMA regressions of wet-fat mass on SVL ranging between 10.21–15.37 (S3 Fig).

**Table 4:** Tests of the assumptions of regression-based body condition indices for different size class groups. Size groups were defined based on the fitted segmented regression model (Fig 2), which defined size groups as hatchlings to early juveniles (group 1, < 20.2 cm SVL), late juveniles to reproductive-sized adults (group 2, 20.2 cm to 30.0 cm SVL) and large adults (group 3, > 30.0 cm SVL). We tested three assumptions, whether 1) data are linear, 2) variance is constant (i.e., homoscedastic), and 3) frequency distribution of residuals is normal. Only p-values are reported.

	Ramsey’s RESET Test (linearity)			Breusch-Pagan Test (homoscedasticity)			Shapiro-Wilk Test (normality)			D’Agostino Test (skewness)			Anscombe-Glynn Test (kurtosis)		
Regression	1	2	3	1	2	3	1	2	3	1	2	3	1	2	3
OLS(logM~logL)	0.567	0.026	0.955	0.992	0.051	0.904	0.059	0.018	0.016	0.019	0.429	0.046	0.664	0.011	0.005
MA(logM~logL)	-	-	-	-	-	-	0.116	0.003	0.027	0.043	0.057	0.081	0.676	0.007	0.025
SMA(logM~logL)	-	-	-	-	-	-	0.071	0.005	0.033	0.024	0.158	0.061	0.682	0.007	0.012
SMA(M~L3)	0.471	0.012	0.632	<0.001	<0.001	<0.001	0.104	<0.001	<0.001	0.113	0.001	0.029	0.031	<0.001	<0.001

Likelihood ratio test for common slopes showed that the allometric relationship between the ln-transformed body mass and ln-transformed SVL did not significantly differ between size groups (p-value = 0.78). The allometric relationship between ln-transformed wet-fat mass and ln-transformed SVL did significantly differ between size class groups (p-value < 0.001). We found some evidence of sex influencing the relationship between ln-transformed body mass and ln-transformed SVL in the group of tegus > 30.0 cm (p-value = 0.02) with a larger slope estimated for female tegus (3.66) than males (3.06), but with overlapping confidence intervals (females 3.20–4.18, males 2.83–3.32).

Residual fat and scaled fat were calculated independently for each size group, because the relationship between wet-fat mass and SVL significantly differed among groups. Residual fat and scaled fat were associated with SVL in each size group, while percent fat was associated with SVL in only the first and second size group (S3 Fig). Of the transformed measures of fat, residual fat exhibited the strongest relationship with SVL in each size group, with adjusted r^2^ values indicating that SVL explains between 26–39% of the variation in residual fat. Both residual fat and scaled fat had negative relationships with SVL in each size group. Relationships between percent fat and SVL were positive but weak in groups 1 and 2, and inexistent in group 3, with SVL explaining approximately 1–3% of the variation in percent fat (S3 Fig).

The testable assumptions of the regression BCIs were still generally not met when data was split into size groups (p-value < 0.05, Table 4). Linearity was rejected for the second size group of tegus for both the ln-transformed linear regression of body mass on length and the regression of body mass on length cubed. Homoscedasticity was rejected for all size groups for the cubed regression index. The normality of residuals was rejected for all BCIs for size groups 2 and 3. Deviations from normality were driven by skewness, kurtosis, or both (Table 4). The slope assumption of Fulton’s K index (slope = 3.0) is violated for group 1 (slope = 3.26, CI 3.13–3.39) and group 2 (slope = 3.22, CI 3.13–3.31), but the estimated slope of group 3 has a 95% confidence interval which overlaps 3.0 (slope = 3.18, CI 2.97–3.39), suggesting that the estimated slope may not represent a significant departure from 3.0.

Percent fat was the only measure of fat stores which was consistently positively associated with measures of body condition across size groups (S2 and S3 Tables in S1 File). When broken down into size groups, significant adjusted r^2^ values from linear regressions of BCIs on fat stores ranged between 0.02–0.26 in group 1, 0.01–0.32 in group 2, and 0.04–0.39 in group 3, suggesting that at best ~ 39% of the variation in BCIs could be explained by fat stores (S2 Table in S1 File). Absolute values of significant Kendall’s correlation coefficient (τ) calculated for BCIs and fat store measures ranged between 0.10–0.39 for group 1, 0.09–0.39 for group 2, and 0.16–0.45 for group 3 (S3 Table in S1 File). Fulton’s K and the OLS residual index were the only BCIs not associated with SVL in each size group when tested with Kendall’s τ (S3 Table in S1 File), though there was a significant relationship predicted between Fulton’s K and SVL predicted by OLS regression in the first size group (S2 Table in S1 File).

Using percent fat as the measure of fat stores, the best performing ratio and residual BCIs across groups were Fulton’s K and the OLS residual index (S2 and S3 Tables in S1 File). However, assumptions of linearity and normality of residuals for the OLS residual index are violated (Table 4), as are assumptions of all other residual indices. The SMI was significantly, positively associated with all measures of fat and, while it is significantly associated with SVL in group 2 and 3, it is a relatively weak association (S2 and S3 Tables in S1 File).

Across size groups, ratio BCIs were more strongly correlated with other ratio indices and residual BCIs were more strongly correlated with other residual indices, with a few exceptions (Fig 3). Fulton’s K index maintained a stronger correlation with residual indices than with other ratio indices. Likewise, the SMI was more strongly correlated with residual BCIs and Fulton’s K than ratio indices. Unlike when all size groups are combined, the cubed regression index maintained strong correlations with other residual indices as well as Fulton’s K and the SMI (Fig 3).

## Discussion

The tegus in our dataset exhibited allometric growth, and we observed generally poor performance of the three measures of body fat and the 11 BCIs we tested against them. None of the measures of body fat were unbiased with respect to SVL, though the variation in percent fat that could be explained by SVL was minimal at 5%. We observed significant weak correlations between the time held between euthanasia and necropsy and our morphometric measurements and BCIs, but believe that the weak strength of these associations makes it unlikely that they would significantly influence our results. It is also possible that these associations were due to the preferential selection of animals for necropsy over time, rather than the direct impact of holding the animals for shorter or longer periods. All regression-based indices violated one or more assumptions, though this improved in some cases after segmenting our dataset into 3 size-class groups. Similarly, BCI performance was somewhat improved after segmenting into the 3 groups, which likely mitigated the effects of allometry to some extent, but the amount of variation in the BCIs that was attributable to percent fat (i.e., ‘true’ body condition) was less than 20%.

Our breakpoint analysis defined three distinct size groups, group 1 is tegus with SVL of < 20.2 cm, group 2 of tegus between 20.2–30.0 cm, and group 3 of tegus > 30.0 cm. We postulate that these size groups roughly align with a first period of growth between hatchling and the first seasonal brumation (group 1), a second period after the first brumation and up to reproductive size adults (group 2), and a third period for adults of reproductive size (> 30.0 cm). Hatchling tegus have been documented with SVL sizes between 7.1 and 8.9 cm [48, 49] and rapidly grow before their first brumation (~ 70 days later) almost doubling in total length [48]. In South Florida, hatching season for tegus occurs between May–June [26] and brumation begins between September and October [50]. This gives hatchling tegus between as much as five months from hatching to first brumation, making it reasonable to expect the first defined size class to consist of tegus at an age just entering their first brumation, or during their second year of life. Female tegus should be able to reproduce by the time they reach 30 cm SVL [48], but a recent study done in South Florida found tegus with gametes at a minimum SVL of 27.0 cm for females and 23.4 cm for males [26]. It is thus reasonable to assume that our second group of tegus represents juveniles and small adults just reaching reproductive age, while the third size group should represent large adults who are almost certainly capable of reproducing.

We found little or no significant differences in the length and mass relationships between sexes which differs from previous studies that found significant differences in SVL between males and females [26] and a difference in the weight-length relationship of adult-sized tegus, but not hatchlings [48]. These differences likely reflect differences in study design. Measurements by Yanosky and Mercolli [48] were taken from 19 males and 12 females during the same year, while our sample size was much larger and spanned multiple years. It is also possible that the introduction of tegu lizards to a novel environment outside of their native range, with abundant food resources, may be affecting morphological or life-history traits of our study population such as body condition, growth rate, and reproduction [51, 52]. Further investigation would be necessary to confirm and identify the mechanisms behind the apparent differences between the South Florida population of tegus and native populations.

In general, the 11 BCIs evaluated explained a relatively low amount of variation in percent fat when evaluated over the whole dataset (r^2^ = 0.05–0.18) and the explanatory power did not significantly improve when evaluated by size classes (r^2^ = 0.06–0.19, S2 Table in S1 File). The OLS residual index was the residual index most strongly associated with percent fat (r^2^ = 0.14) and Fulton’s K was the ratio index most strongly associated with percent fat (r^2^ = 0.18), and this finding was consistent when data were analyzed by size class (S2 Table in S1 File). When analyzing the full dataset, all BCIs, excluding the OLS residual index, were size-biased, increasing as SVL increased. When data were analyzed as smaller size groups, neither Fulton’s K nor the OLS residual index were size-biased. Thus, Fulton’s K index and the OLS residual index were the best performing BCIs.

We found that, in general, ratio BCIs were more strongly correlated with other ratio BCIs as compared to residual indices, except for Fulton’s index, which was more strongly correlated with residual BCIs. Similarly, residual indices were more strongly correlated with other residual indices and the SMI compared to ratio indices. In the evaluation of the full dataset the cubed regression index was relatively weakly correlated with other residual indices, but this was resolved when evaluating smaller size class groups and the strength of the relationship was more comparable to that of the other residual indices. These patterns altogether in tegus suggest that, apart from Fulton’s index, ratio indices are substitutable for one another, and residual indices are generally substitutable with one another or with SMI. Because Fulton’s index is relatively strongly correlated to the residual indices tested here, it is the most substitutable ratio index for any of the residual indices tested. However, we caution against substitution without first evaluating the association of the intended substitute with size (e.g., SVL) and without consideration of their inherent statistical assumptions.

Due to the assumptions of BCIs, different indices may generate different results when used on the same populations or make their use inappropriate for certain applications [11]. Due to the nature of their calculation, residual indices are not comparable across populations. If residual indices are calculated for size classes within a population individually, as we have, comparisons should also not be made between size classes. This complication with using size classes also arises with ratio indices that use slopes, such as the relative index [11], and the SMI index, though in this case it may be utilized if a standardized L_0_ is set for all size classes [20]. Furthermore, our dataset generally violated the assumptions of residual indices, whether the data were split into size classes or not (Tables 3 and 4), limiting the conclusions that can be drawn when using these BCIs to evaluate body condition.

Ratio indices are comparable across populations but are often associated with body size [11]. Fulton’s index was the best-performing ratio index for our dataset, but the slope estimates for our full dataset and size class analyses violate the assumption that the slope of the relationship between ln-transformed mass and ln-transformed SVL is equal to exactly 3.0 [4]. If Fulton’s index is used on a population with a slope significantly greater than 3.0, the index may be positively associated with length, causing issues with comparability between populations, or within a single group with a large range of body sizes. Because our dataset violates assumptions of all the residual BCIs tested, and because a ratio BCI may be used to make comparisons between groups, we determined that Fulton’s index was the best-performing BCI overall, as it was the most associated with percent fat and least-biased with respect to body size. However, care should be taken to validate the slope assumption and determine how violations may impact study findings.

While analyzing data in size class groups reduced the association of percent fat with SVL and reduced the association of Fulton’s K with SVL, it did not generally improve BCI performance. The weak association between BCIs and fat in tegus could be attributed to several factors. First, it appears that tegus violate a basic assumption of many BCIs; that measures of fat scale isometrically with size [41]. Indeed, de Souza et al. [25] determined that there was disproportional deposition of fat by larger individuals of the same age, with larger tegus depositing significantly more lipids prior to their first brumation [25]. The violation of the assumption of the independence of fat stores and body size may occur if larger animals have improved access to food resources [18]. Size-dependence of fat stores and improved access to food resources may be indicated by the reduced association of percent fat with SVL when the data were analyzed by size class (S3 Fig). Because of this size-dependent relationship, we suggest that future studies considering body condition in South Florida tegus should focus on defined size-related groups, and that when making comparisons between populations they should be made between the same size-classes. Future work could be conducted to study how the growth rate of tegus within South Florida changes with respect to SVL to validate our assigned size classes or improve size class assignment.

Additionally, tegus have seasonal metabolic and hormonal cycles which drive behavioral and physiological changes in energy storage and energy use [25, 26, 53]. Hence, the mass of individuals and their abdominal fat bodies regularly cycle throughout the year. Percent fat of tegus in South Florida, calculated using the mass of coelomic fat deposits, was previously found to cycle relative to periods of brumation and reproduction [26]. Because we obtained animals in all months of the year in which they were active, and did not separate the data by month, there will be variation in percent fat and body weight between animals of comparable sizes due to these normal physiological cycles. This variation may have influenced the strength of the relationship detected between the BCIs tested and percent fat. This source of variation could be accounted for by changing sampling designs to compensate for regular seasonal changes in fat mass when attempting to draw conclusions about relative condition. Using multiple years of data may additionally complicate BCI analyses if conditions and resource availability change annually [11].

It is also possible that the coelomic wet-fat mass is not a good representation of the total energy storage available to tegus. The difference in the body plan of tegus compared to snakes provides alternative locations for lipid storage, such as the tail [23, 54]. Tegus are known to store lipids within the tail and the liver and to rely on these stores, as well as abdominal fat bodies, during brumation [23, 25]. Because we did not account for total mass of fat stored outside of coelomic fat bodies, a different approach which quantifies fat throughout the body (e.g., desiccating and grinding the specimen and then measuring fat) may produce better results. Warner et al. [55] attempted to validate BCIs in the small-bodied lizard, *Anolis sagrei*, using quantitative magnetic resonance and chemical carcass analysis to determine whole-body composition (i.e., lean muscle, fat, and water content). However, despite accounting for total fat content, the authors found weak or insignificant positive relationships between the BCIs evaluated (OLS residual index and SMI) and percent fat [55]. These results, combined with our findings, highlight the need to proceed with caution when interpreting patterns in body condition of lizards when solely utilizing BCIs.

Other types of tissue which would not be accounted for by our methods, such as muscle tissue, may be better related to fitness in South Florida tegu lizards. Seasonal weight loss in tegus has been attributed to the loss of skeletal tail muscle during brumation [25], indicating that muscle tissue in the tail is relied on during at least part of the year as an energetic reserve and that fat mass alone is an insufficient measure of body condition. We also assume that the measure of length being used to calculate body condition is a good measure of overall size, but if there is a large amount of variation in shape between individuals of a given size it is possible that SVL is not adequately capturing these changes in shape [12]. For example, sexually mature male tegus have larger jaw muscles, visible as large jowls, and increased head mass compared to sexually mature female tegus, the mass of which also varies seasonally with reproduction [56]. Further work could clarify whether both BCIs and aspects of tegu biology (e.g., reproductive output or stress levels) are more strongly associated with fat, muscle, or another kind of tissue.

Alternatively, different measures of fitness, such as plasma corticosterone or reproductive output, may be more closely related to BCIs in tegus than measures of fat. For example, levels of plasma corticosterone are often used as an indicator of chronic stress in a population and have been correlated with BCIs; individuals with low index values tend to have high levels of plasma corticosterone [57]. A study of adult captive-bred tegus in Brazil found that plasma corticosterone concentrations changed seasonally, likely based on reproductive cycles and brumation [58], and indeed these trends seemed to roughly follow seasonal cycles of fat found in South Florida tegus [26]. In another example, Litzgus et al. [59] found a positive relationship between body condition and clutch mass and egg size in spotted turtles. Using a combination of corticosterone, clutch mass, and fat mass may serve as a more informative measure of fitness in tegus than using either measure on its own [57]. Studies of survival could help determine if there may be traits under more consistent selection to better represent true body condition [60].

Despite the methodological and statistical issues described above, BCI’s will continue to be widely used by ecologists for conservation purposes [8]. However, in many cases these indices have not been empirically validated in the population under study and hence, should be used cautiously. Preferably, anyone using a morphometric BCI should validate that index for the population, sex, size class (age group), season of interest [2], and hypothesis to be tested. When a BCI has not been validated for the population being studied, we suggest including the morphometric variables that are hypothesized to be relevant for condition directly, such as percent fat, corticosterone, or other measures of fitness such as clutch mass, in the analysis [2, 8]. Using a combination of different classes of BCIs or levels of biological organization may also be advantageous [8, 11]. For tegu lizards in South Florida, we determined that percent fat served as the best measure of fat for validating BCIs, although alternative measures of fitness should be assessed as alternative or complimentary validation metrics. We identified Fulton’s index as the best-performing and least size-biased BCI of the 11 tested, although care should be taken when comparing groups with different mean body sizes, or with significantly different relationships between body mass and length.

## Supporting information

S1 Fig A comparison of the basic allometric model (black line) to the segmented linear regression (red line) with the mean weight of tegus, grouped in 2.0 cm groups. Mean weights are plotted with error bars representing the standard error of each group. The 95% confidence intervals of model estimates are represented by shaded areas surrounding the mean estimate line. Zoomed-in plots (B, C, D) are provided to better examine the intersection of the predicted model values with group mean weights.
(TIF)

S2 Fig The ln-transformed snout-vent length (SVL) and ln-transformed body mass of tegus, with colors representing assigned size classes. (TIF)

S3 Fig Relationships between ln-transformed relationships between snout-vent length (SVL) and body mass, fat mass, and three measures of fat stores for size group 1 (A–E), size group 2 (F–J), and size group 3 (K–O). SMA regression equation intercepts and slopes, and adjusted r2 values are reported to two significant digits. Significance values are reported as * P ≤ 0.05, ** P ≤ 0.01, and *** P ≤ 0.001.
(TIF)

S1 File Contains S1-S3 Tables. (XLSX)
