# Correction: How to estimate body condition in large lizards? Argentine black and white tegu (*Salvator merianae*, Duméril and Bibron, 1839) as a case study

**DOI:** 10.1371/journal.pone.0307985

**Published:** 2024-07-25

**Authors:** Kelly R. McCaffrey, Sergio A. Balaguera-Reina, Bryan G. Falk, Emily V. Gati, Jenna M. Cole, Frank J. Mazzotti

There is an error in reference 50. The correct reference is: McEachern MA, Yackel Adams AA, Klug PE, Fitzgerald LE, Reed RN. Brumation of introduced black and white tegus, *Tupinambis merianae* (Squamata: Teiidae), in southern Florida. Southeast Nat. 2015;14(2): 319–328.

In Fig 2, there is an error in the third equation. The symbol “<” should have been “>”. The correct equation should read as “log(y) = 4.313 + 0.083x, x > 30.0”. Please see the correct Fig 2 here.

**Fig 2:**
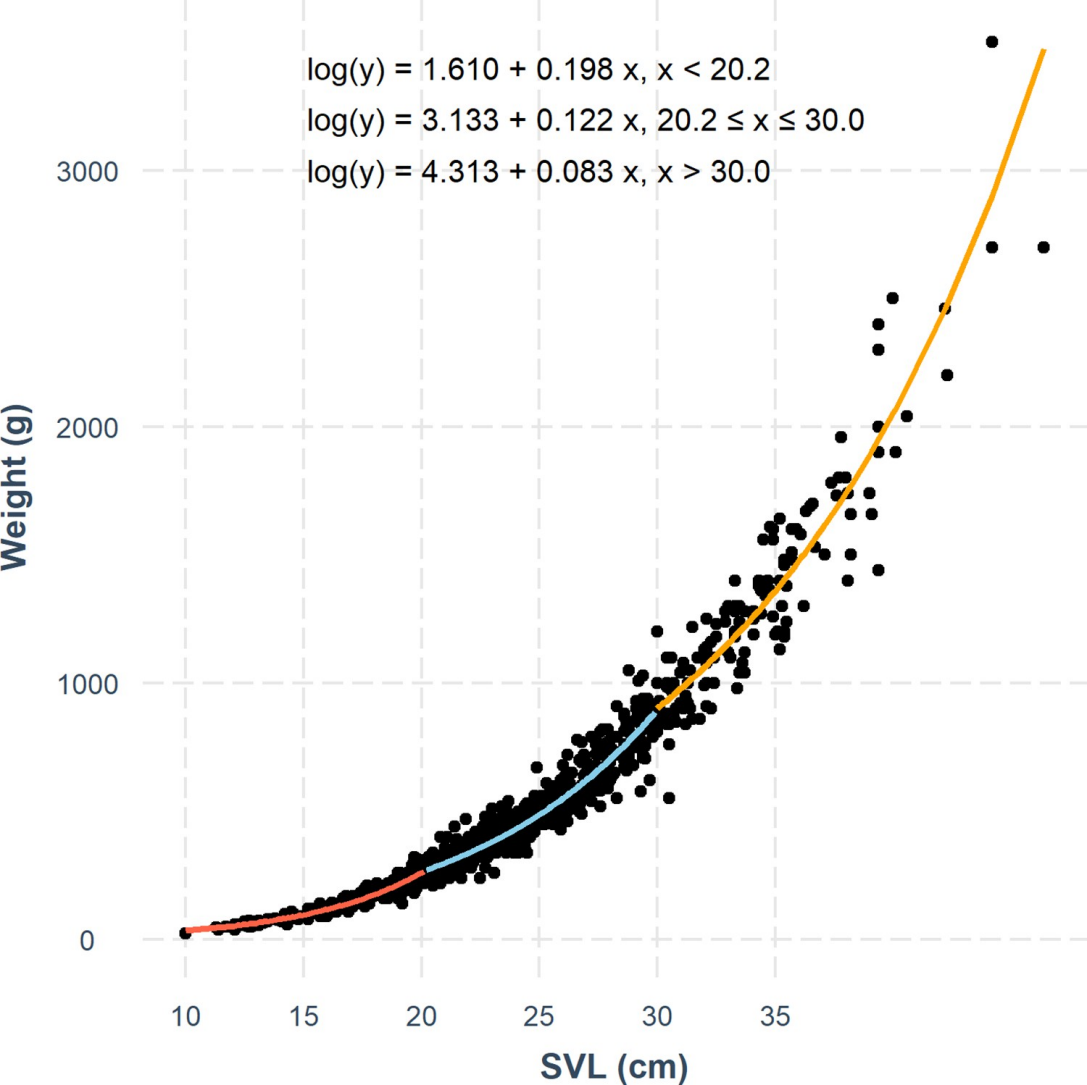
The three-segmented generalized linear model fit to the relationship between snout-vent length (SVL) and total mass, splitting the data into three size groups: Hatchlings to early juveniles (group 1, < 20.2 cm), late juveniles to reproductive-sized adults (group 2, 20.2 cm– 30.0 cm) and large adults (group 3, > 30.0 cm).
