# Supplementary figures and images for: How to estimate body condition in large lizards? Argentine black and white tegu (Salvator merianae, Duméril and Bibron, 1839) as a case study
Source: PLoS One. 2023 Feb 24;18(2):e0282093. doi: 10.1371/journal.pone.0282093 (PMC9955610; doi:10.1371/journal.pone.0282093)

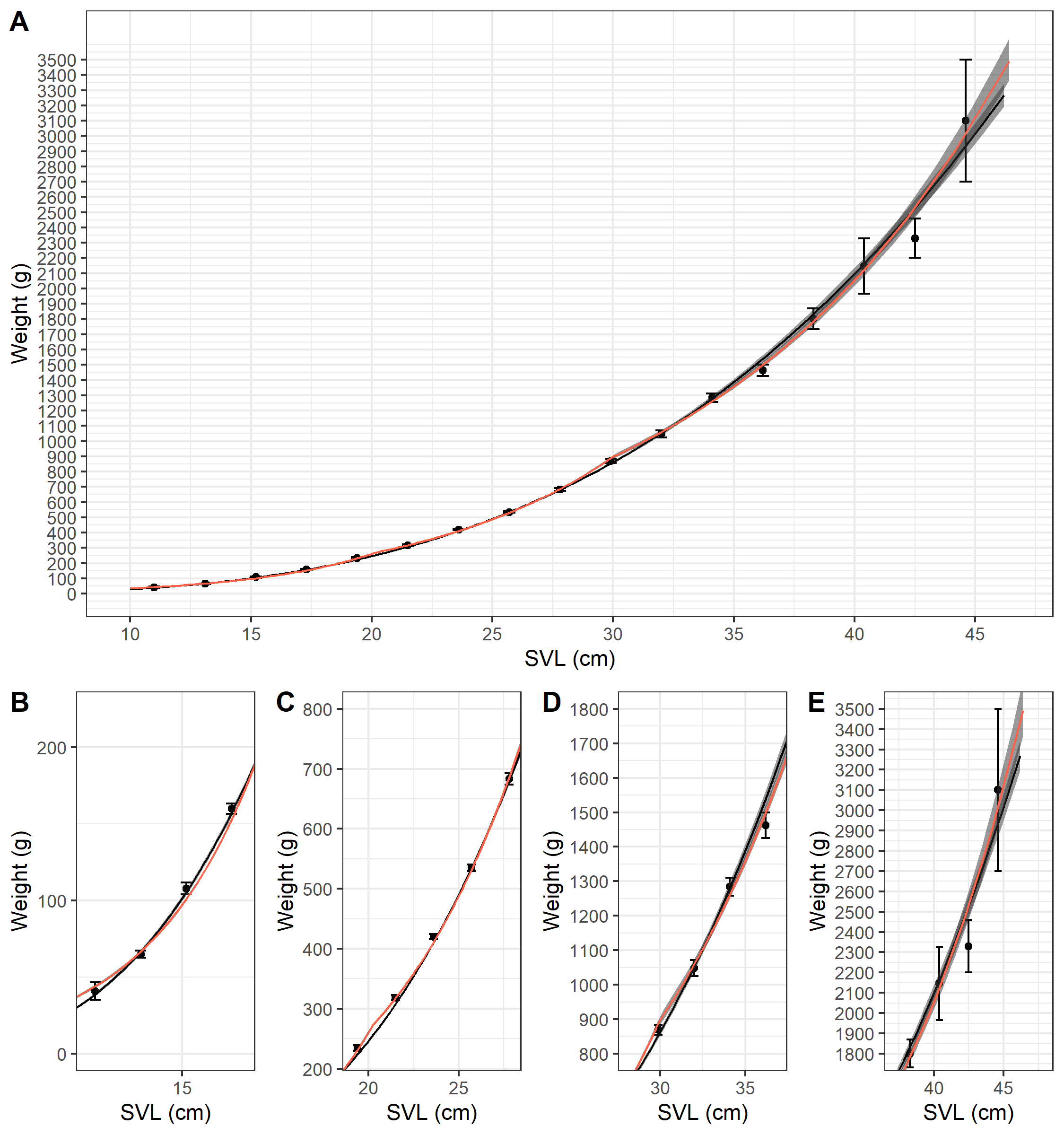

Supplement: S1 Fig — Mean weights are plotted with error bars representing the standard error of each group. The 95% confidence intervals of model estimates are represented by shaded areas surrounding the mean estimate line. Zoomed-in plots (B, C, D) are provided to better examine the intersection of the predicted model values with group mean weights. (TIF) [file pone.0282093.s001.tif]

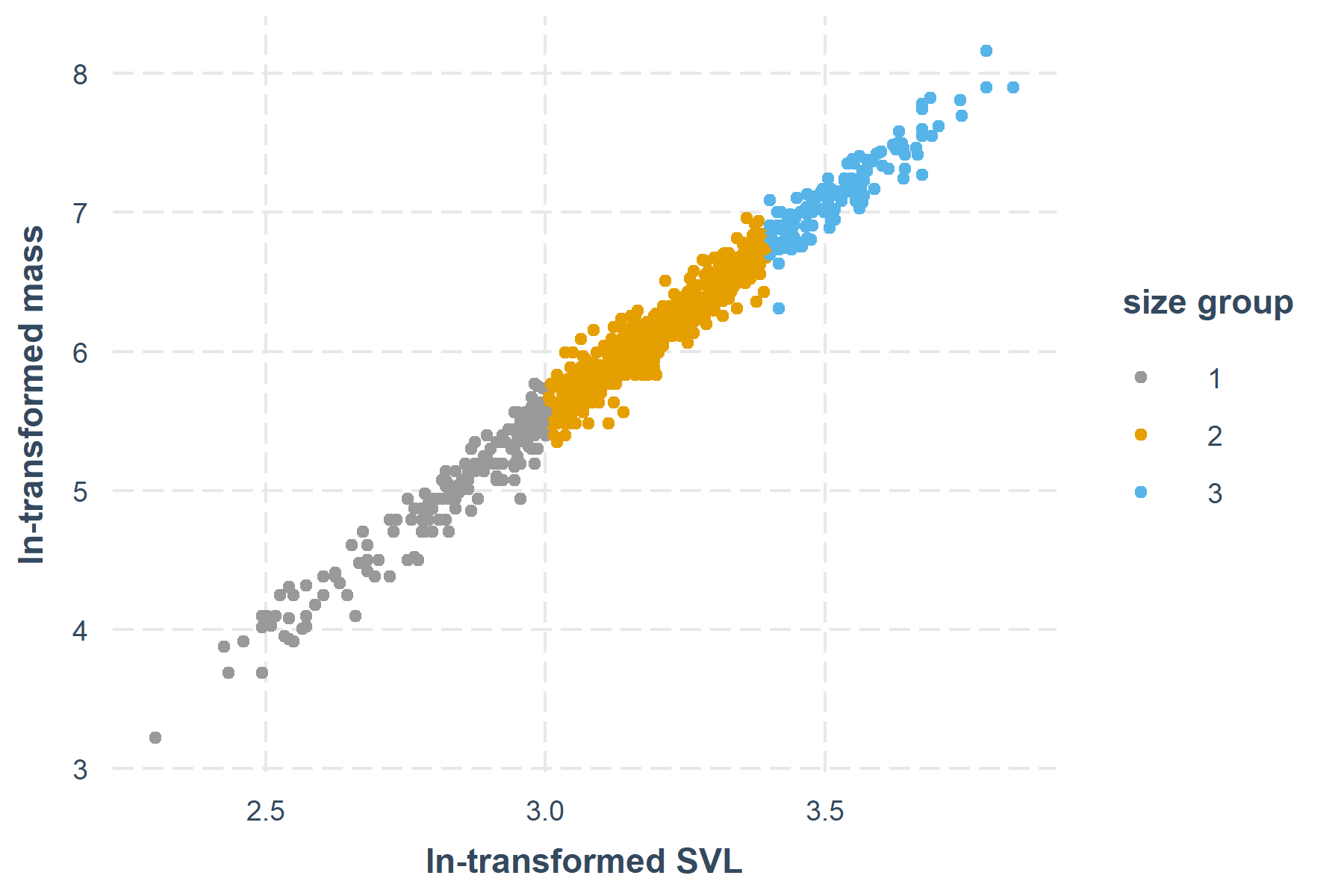

Supplement: S2 Fig — (TIF) [file pone.0282093.s002.tif]

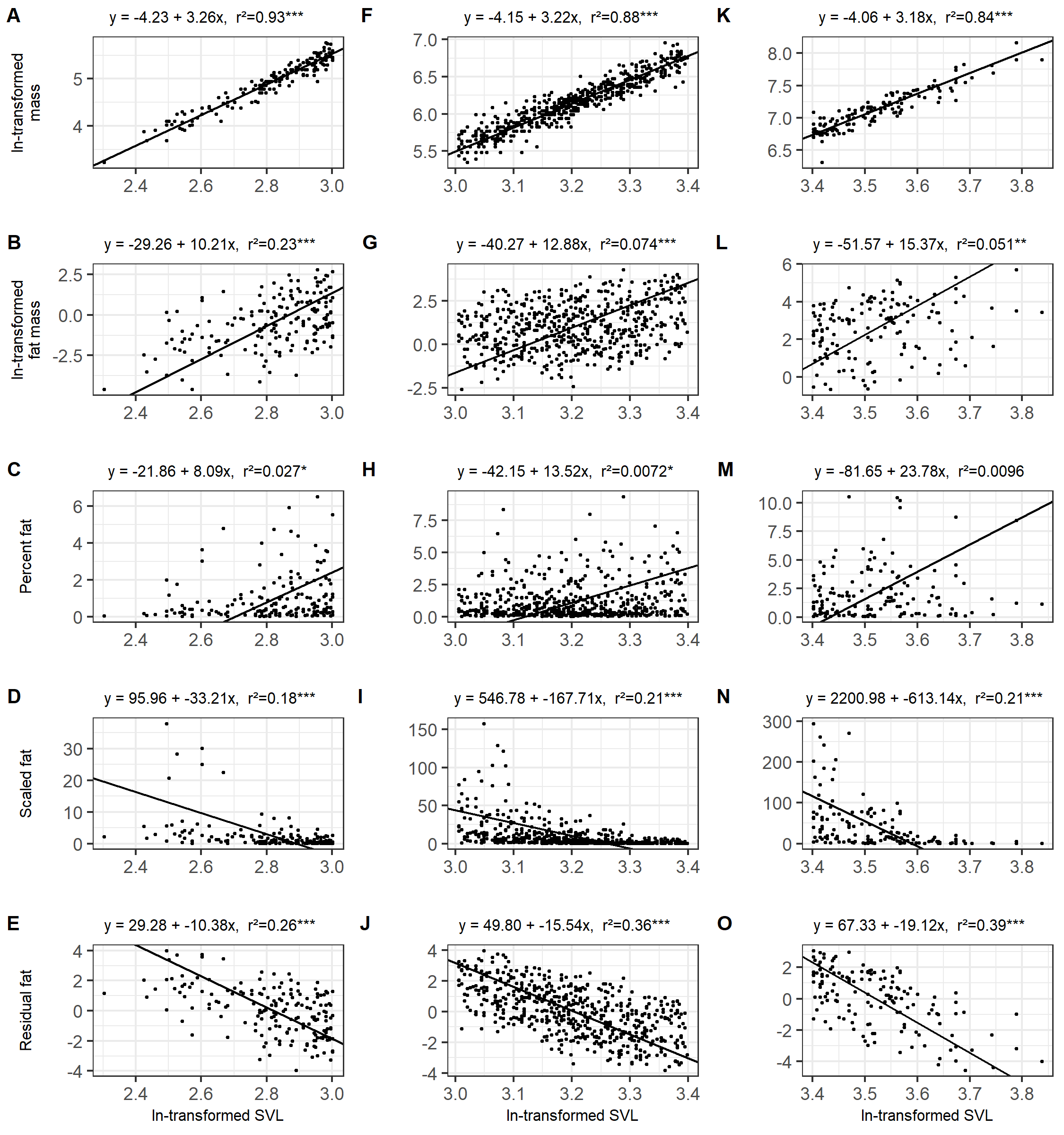

Supplement: S3 Fig — Relationships between ln-transformed relationships between snout-vent length (SVL) and body mass, fat mass, and three measures of fat stores for size group 1 (A–E), size group 2 (F–J), and size group 3 (K–O). SMA regression equation intercepts and slopes, and adjusted r2 values are reported to two significant digits. Significance values are reported as * P ≤ 0.05, ** P ≤ 0.01, and *** P ≤ 0.001. (TIF) [file pone.0282093.s003.tif]
